# The laws and effects of terahertz wave interactions with neurons

**DOI:** 10.3389/fbioe.2023.1147684

**Published:** 2023-04-26

**Authors:** Ma Shaoqing, Li Zhiwei, Gong Shixiang, Lu Chengbiao, Li Xiaoli, Li Yingwei

**Affiliations:** ^1^ School of Information Science and Engineering, Yanshan University, Qinhuangdao, China; ^2^ Hebei Key Laboratory of Information Transmission and Signal Processing, Qinhuangdao, China; ^3^ Institute of Electrical Engineering, Yanshan University, Qinhuangdao, China; ^4^ Henan International Key Laboratory for Noninvasive Neuromodulation, Xinxiang Medical University, Xinxiang, China; ^5^ State Key Laboratory of Cognitive Neuroscience and Learning, Beijing Normal University, Beijing, China

**Keywords:** terahertz waves, neurons, dynamic growth, cumulative radiation, thermal effect

## Abstract

**Introduction:** Terahertz waves lie within the energy range of hydrogen bonding and van der Waals forces. They can couple directly with proteins to excite non-linear resonance effects in proteins, and thus affect the structure of neurons. However, it remains unclear which terahertz radiation protocols modulate the structure of neurons. Furthermore, guidelines and methods for selecting terahertz radiation parameters are lacking.

**Methods:** In this study, the propagation and thermal effects of 0.3–3 THz wave interactions with neurons were modelled, and the field strength and temperature variations were used as evaluation criteria. On this basis, we experimentally investigated the effects of cumulative radiation from terahertz waves on neuron structure.

**Results:** The results show that the frequency and power of terahertz waves are the main factors influencing field strength and temperature in neurons, and that there is a positive correlation between them. Appropriate reductions in radiation power can mitigate the rise in temperature in the neurons, and can also be used in the form of pulsed waves, limiting the duration of a single radiation to the millisecond level. Short bursts of cumulative radiation can also be used. Broadband trace terahertz (0.1–2 THz, maximum radiated power 100 μW) with short duration cumulative radiation (3 min/day, 3 days) does not cause neuronal death. This radiation protocol can also promote the growth of neuronal cytosomes and protrusions.

**Discussion:** This paper provides guidelines and methods for terahertz radiation parameter selection in the study of terahertz neurobiological effects. Additionally, it verifies that the short-duration cumulative radiation can modulate the structure of neurons.

## Introduction

Terahertz waves are electromagnetic waves located between microwave and far infrared with a frequency range between 0.1–10 THz. Terahertz waves are in the energy range for hydrogen bonding, charge transfer reactions, and van der Waals forces, suggesting that many of the rotational, oscillatory, torsional, and other energy levels of biological macromolecules (proteins, DNA, RNA) are only in the terahertz band (Alexandrovet et al., 2009; Cherkasova et al., 2009; Wang et al., 2018; Sun et al., 2021). Thus, terahertz waves of specific frequencies and energies can be coupled directly to proteins to induce coherent excitation to produce non-thermal effects (Bo et al., 2021; Peng and Zhou, 2021). For example, terahertz radiation interacts with hydrogen bonds in proteins (Fischer et al., 2002), causing low-frequency molecular vibrations that lead to changes in the conformation and functional characteristics of the protein (Cherkasova et al., 2009). It can also cause non-thermal structural changes in protein crystals (Lundholm et al., 2015). Some algorithms can also be used to improve the spectral quality when performing spectral analysis, such as the KLT transform as described by Zaharov et al. (2014). Through computer simulation modeling research, Alexandrov et al. (2009) found that terahertz radiation may interfere with the naturally occurring local strand separation kinetics of double-stranded DNA, thereby affecting the function of the DNA. The study points out that the main effect of terahertz radiation is that resonance affects the dynamical stability of the dsDNA system. It has also been shown that terahertz radiation can precisely control the proton transfer process in the hydrogen bonding of base pairs, and can control DNA demethylation (Cheon et al., 2019a; Cheon et al., 2019b; Wang et al., 2020). These studies suggest that terahertz waves can mediate changes in cell structure and function by exciting non-linear resonance effects in proteins and DNA. Based on this mechanism, terahertz waves of specific frequencies and energies affect the structure and function of neurons.

Currently, many scholars are beginning to focus on neurons’ response to terahertz waves, but it is important to consider the safety of terahertz radiation protocols. Although terahertz waves are low in energy and do not ionize matter, this does not mean that they are safe (Yang et al., 2016; Mancini et al., 2022). Several studies have shown that terahertz waves’ effects on neurons are two-fold. For example, terahertz radiation (3.68 THz, 10–20 mW/cm^2^, 60 min) causes neuronal growth disorders (Olshevskaya et al., 2009). When the terahertz radiation power was further increased (2.1 THz, 30 mW/cm^2^, 1 min), it resulted in a decrease in neuronal membrane potential with morphological disturbances and death after 2 h of radiation (Olshevskaya et al., 2010). When the radiation power was reduced to 3 mw/cm^2^, neuron death occurred 3 h after radiation. Continuous wave terahertz radiation (0.12–0.18 THz, 3.2 mw/cm^2^, 1–5 min) also leads to increased neuronal mortality (Borovkova et al., 2016). The above studies show that high power, continuous waves and prolonged radiation have many negative effects on neurons. However, several studies have shown that terahertz radiation has positive effects on the structure and function of neurons. The growth of neuronal protrusions was promoted when neurons were radiated using broadband micro-terahertz (0.05–2 THz, 50 uW/cm^2^, 3 min). This promotion persisted when the power was reduced to 0.5 uW/cm^2^ (Tsurkan et al., 2012). Additionally, narrowband trace terahertz (0.06 THz, 40–840 nW, 1 min) increased the neurons’ firing rate (Pikov et al., 2010). It has also been noted that terahertz radiation has no significant effect on either neuronal activity or survival (Sun et al., 2021; Zhao et al., 2021). These phenomena indicate the non-linearity of terahertz neurobiological effects. The reason for these phenomena is associated with the total power radiated to the neuron by the terahertz, where the total power is related to the terahertz wave’s frequency, energy and radiation time (Bo et al., 2021; Chen et al., 2022).

Terahertz radiation safety parameters should both ensure sufficient power radiation to the neuron and reduce the temperature effect on the neuron. This is a dilemma, and choosing the appropriate terahertz radiation parameters can mitigate the negative effects of terahertz waves on neurons (Hu et al., 2021). However, there is still a lack of guidelines and methods for selecting terahertz radiation parameters. Current research has identified positive effects of terahertz radiation on neuronal structure and function. For example, terahertz radiation can alter neuronal growth and development-related gene expression kinetics, and promote the growth of neuronal protrusions (Sun et al., 2021; Zhao et al., 2021). However, neuronal growth and development is a dynamic, ongoing process, and there are no studies which point to the short- and long-term effects of terahertz radiation on dynamic neuronal growth.

In this study, the propagation and thermal effects of terahertz wave interactions with neurons were modelled to analyze the main parameters affecting the field strength and temperature changes of terahertz waves in neurons. The field strength and temperature variations are used as evaluation criteria to select the appropriate terahertz radiation parameters. On this basis, we experimentally investigated the effect of cumulative radiation from Terahertz waves on neuronal structures. First, we built a broadband micro terahertz radiation system (0.1–2 THz, maximum radiation power 100 μW) and measured the absorption of terahertz waves by neuronal culture dishes and culture fluid. Secondly, we cultured SD rat primary cortical neurons *in vitro* and recorded the dynamic growth of neurons using broadband micro terahertz waves to focus radiation on cortical neurons for a short period. Finally, we analyzed the effect pattern of broadband trace terahertz radiation on neuronal cytosol and protrusion.

## Materials and methods

### The geometry of a neuronal electromagnetic-thermal coupled multiphysics field model

**FIGURE 1:**
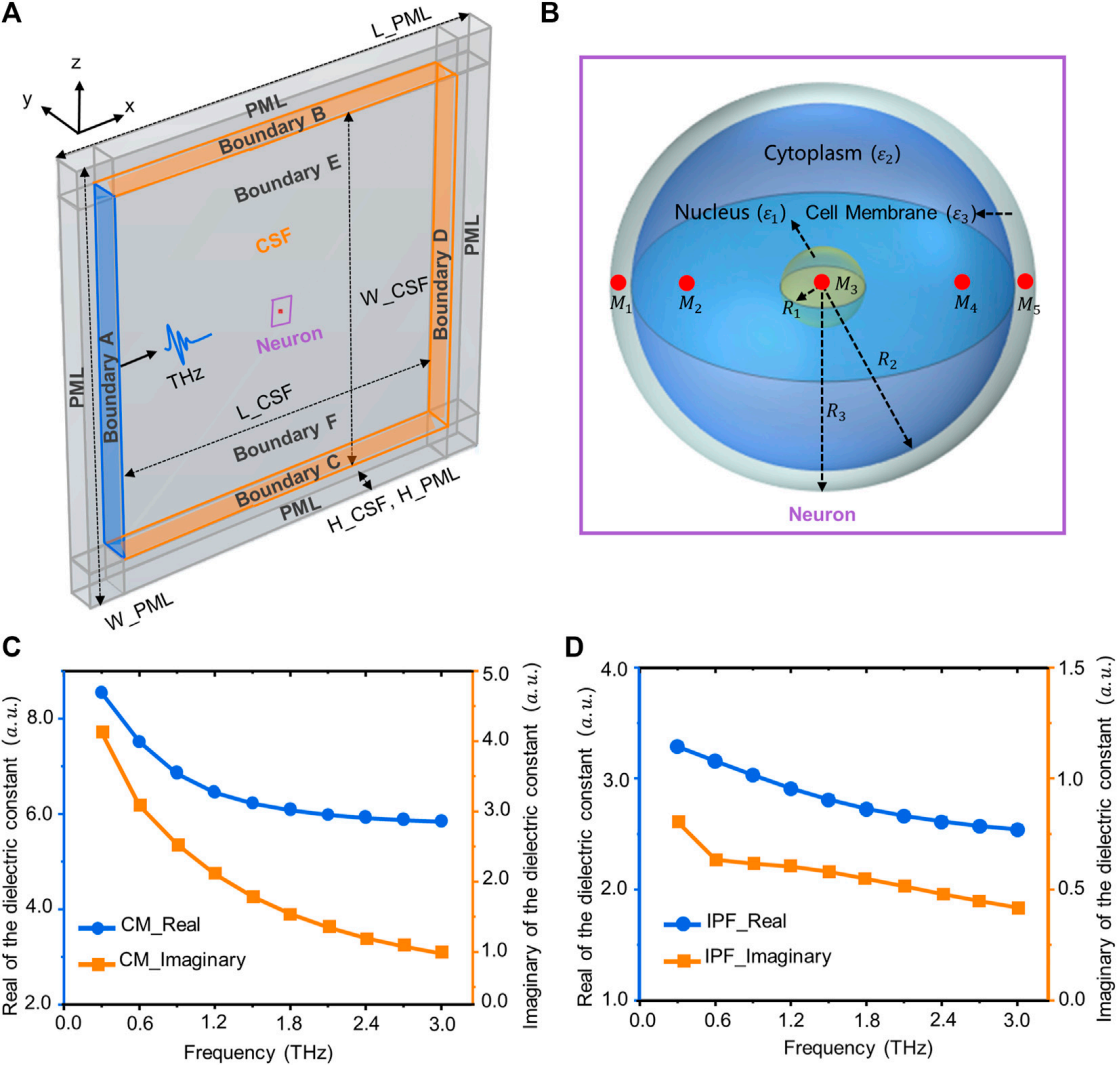
Simulation model and dielectric constant of biological tissue. **(A)** Terahertz wave transport and thermal effect model in the neuron, including neurons, terahertz sources, cerebrospinal fluid, and PML layers. **(B)** A three-dimensional neuronal model consisting of a nucleus, cell membrane (CM) and cytoplasm. The red area is the location of the sampling point (M_1_–M_4_). **(C)** The real and imaginary parts of the relative permittivity of the CM (3–3 THz). **(D)** The real and imaginary parts of the relative permittivity of the intracellular physiological fluid (IPF).

We first constructed the geometry of the neuronal electromagnetic-thermal coupled multiphysics field. The entire simulation area is a cuboid, containing terahertz sources, the perfect matching layer (PML), neurons, and cerebrospinal fluid (Figure 1A). The size of the model is shown in Table 1. Depending on the frequency range of the simulation, we use the RF module to simulate a terahertz source. The incident port of the terahertz wave is shown in the blue area in Figure 1A. Terahertz waves first enter the cerebrospinal fluid. After they have travelled a distance of approximately 10 um in the cerebrospinal fluid, they enter the neuron. Finally the terahertz waves are absorbed in their entirety by the PML layer.

**TABLE 1:** Dimensions of the simulation domain geometry.

Region	Description	Parameter	Value
CSF	The length of the CSF domain	L_CSF	2.5 mm
	The width of the CSF domain	W_CSF	2.5 mm
	The height of the CSF domain	H_CSF	0.15 mm
Neuron	The nuclear radius of the neuron	R_1_	1.0 um
	The thickness of neuron’s cytoplasm	R_2_–R_1_	8.0 um
	The thickness of neuron’s cell membrane	R_3_–R_2_	1.0 um
PML	The length of the PML domain	L_PML	3.0 mm
	The width of the PML domain	W_PML	3.0 mm
	The height of the PML domain	H_PML	0.15 mm

Neurons are the most basic structural and functional units in neural networks. They contain the protrusions and cytosol (Ajenikoko et al., 2023). The cell body consists of the nucleus, the cell membrane and the cytoplasm, while the protrusions are divided into dendrites and axons. The morphology of a neuron affects the way it receives and transmits information, and varies according to its function (Ma et al., 2023). Common neuronal cytosol morphologies include star-shaped, conical, ellipsoidal and round, and the number of protrusions on the cytosol varies (Yu et al., 2023). This is due to the wide variety of neurons and the difficulty of measuring the dielectric constants of individual neurons. Therefore, for this paper, we have simplified the structure of the neurons, and established a circular neuron model with a three-layer structure containing the cell membrane, cytoplasm and nucleus (Figure 1B). The red areas in the figure are the locations of sampling points, which are used to analyze the distribution of terahertz waves in different regions of neurons. The dimensions of the neuron geometry are shown in Table 1.

### Neurons’ relative dielectric constants

The neuronal membrane consists of a phospholipid bilayer that forms the basic cytoskeleton, with a variety of receptors and ion channels capable of receiving and transmitting excitation (Di Cristo and Chattopadhyaya, 2020). Due to the complexity of the membranes and the difficulty of ensuring their intact structure during extraction, the relative permittivity of phospholipids was used to characterize the relative permittivity of neuronal membranes. The relative permittivity of phospholipids in the terahertz band can be obtained from the literature (Paparo et al., 2009). After fitting through the second order Debye model, the Debye parameters are shown in Table 2. The fitted parameters were brought into Eq. 1 (Guo et al., 2021) to obtain the relationship between the relative permittivity of the neuronal membrane and frequency (Figure 1C).

$$\hat{\varepsilon }\left(\omega \right)=\varepsilon_{\infty }+\frac{\varepsilon_{s}-\varepsilon_{2}}{1+i\omega \tau_{1}}+\frac{\varepsilon_{2}-\varepsilon_{\infty }}{1+i\omega \tau_{2}}$$ (1)

where ω is angular frequency; $\varepsilon_{\infty }$ is high frequency limit relative dielectric constant; $\varepsilon_{s}$ is static relative dielectric constant; $\varepsilon_{2}$ is the median value; $\tau_{1}$ and $\tau_{2}$ are relaxation times. The slow relaxation time $\tau_{1}$ is related primarily to hydrogen bonding, which reflects the change from $\varepsilon_{s}$ to $\varepsilon_{2}$ . The fast relaxation time $\tau_{2}$ is related to the reorientation of individual molecules, which reflects the change from $\varepsilon_{2}$ to $\varepsilon_{\infty }$ .

**TABLE 2:** Results of fitting the parameters of the second-order Debye model for cell membrane and intracellular physiological fluid.

Type	$\varepsilon_{\infty }$	$\varepsilon_{S}$	$\varepsilon_{2}$	$\tau_{1}/ps$	$\tau_{2}/ps$
Cell membrane	2.37	11.77	3.28	7.19	0.11
Intracellular physiological fluid	5.69	77.34	8.99	12.60	0.24

The solvent molecules in the nucleus are the same as those in the cytoplasm. Therefore, the intracellular physiological fluid’s relative permittivity is used to represent the nucleus’ relative permittivity. In addition, the radius of the organelles such as mitochondria is about 1 um, much smaller than the terahertz wavelengths examined for this paper (100–1 mm). Thus, tiny organelles exhibit only diffraction and bypassing behavior, and therefore their effects are also neglected in the calculations. The relative permittivity of the physiological fluids within neurons can be obtained from the literature (Guo et al., 2021). After fitting through the second-order Debye model, the Debye parameters are shown in Table 2. The relationship between the relative dielectric constants of physiological fluids and frequency are shown in Figure 1D.

### Calculation method for the transmission characteristics for terahertz waves in neurons

In the simulation, we use the finite element method to solve the system of Maxwell’s equations in full wave form, where the partial differential form of Maxwell’s equations is:

$$\left\{\begin{array}{l} \nabla ∙D=\rho \\ \begin{array}{l} \nabla \times E=-\frac{\partial B}{\partial t}\\ \begin{array}{l} \nabla ∙B=0\\ \nabla \times H=J+\frac{\partial D}{\partial t} \end{array} \end{array} \end{array}\right.$$ (2)

In the formula, E is the strength of the electric field ( V/m ); H is the magnetic field strength ( A/m ); D is the electric flux density ( $C/m^{2})$ ; B is the magnetic flux density ( $Wb/m^{2}$ ); J is the current density ( $A/m^{2}$ ).

In each isotropic medium, the intrinsic relations of the electromagnetic field are:

$$\left\{\begin{array}{c} D=\varepsilon E\\ \begin{array}{c} B=\mu H\\ J=\sigma E \end{array} \end{array}\right.$$ (3)

where ε is the dielectric constant ( F/m ); μ is the magnetic permeability ( H/m ); σ is the electrical conductivity ( S/m ). In the solution process, it is assumed that the angular frequency is known: ω=2πf . The speed of light in a vacuum is $c_{0}$ . The electromagnetic field varies sinusoidally with time, and all properties of the materials are linear with respect to the field strength. Then the steady-state form of the three-dimensional Maxwell control equations can be simplified as:

$$\nabla \times \left({\mu_{r}}^{-1}\nabla \times E\right)-{k_{0}}^{2}\left(\varepsilon_{r}-\frac{j\sigma }{\omega \varepsilon_{0}}\right)E=0$$ (4)

$$k_{0}=\frac{\omega }{c_{0}}$$ (5)

where $\mu_{r}$ is the relative magnetic permeability; $\varepsilon_{r}$ is the relative dielectric constant; σ is the electrical conductivity. For steady state analysis, we solved the above equations for the electric field $E=E\left(x,y,z\right)$ over the entire simulation area, where E is a vector and its weight is $E=E\left(E_{x},E_{y},E_{z}\right)$ . All other physical quantities, including the magnetic field, current and power, can be deduced from the electric field.

In solving Maxwell’s steady state equations along three dimensions, the boundary B-F is set to a scattering boundary condition (Figure 1A). In addition, we add PML layers to the outermost layer of the simulation domain to absorb electromagnetic waves (Figure 1A). Setting the boundary A as a terahertz source produces a terahertz wave in sinusoidal form which propagates along the *x*-axis (Figure 1A). The output parameters of the terahertz source, the initial conditions for solving the equations, and the properties of the materials are shown in Table 3.

**TABLE 3:** The initial conditions for solving the equations and the properties of the materials.

Type	Description	Value	References
Terahertz source	Frequency	f=0.3THz	
	Power	P=1mW	
Initial temperature	Temperature	$T_{0}=298.15\;K$	
Neuron membrane	Relative dielectric constant	3.28 + 0.801i a.u.	Paparo et al. (2009)
	Relative permeability	1 a.u.	
	Specific conductance	0 S/m	
Neuron Intracellular physiological fluid	Relative dielectric constant	8.53 + 4.12i a.u.	Guo et al. (2021)
	Relative permeability	1 a.u.	
	Specific conductance	0 S/m	
Neuronal thermal parameter	Thermal conductivity	$k=0.49\;W/\left(m∙K\right)$	ITIS (2023)
	Density	$\rho =1075\;kg/m^{3}$	
	Constant pressure specific heat capacity	$C_{p}=3613\;J/\left(kg∙K\right)$	
cerebrospinal fluid (CSF)	Relative dielectric constant	5.34 a.u.	ITIS (2023)
	Relative permeability	1 a.u.	
	Specific conductance	92.7 S/m	
	Thermal conductivity	$k=0.57\;W/\left(m∙K\right)$	ITIS (2023)
	Density	$\rho =1007\;kg/m^{3}$	
	Constant pressure specific heat capacity	$C_{p}=4096\;J/\left(kg∙K\right)$	

### Calculation method for the thermal effects of terahertz wave interactions with neurons

When terahertz waves radiate a neuron, the neuron absorbs a certain amount of terahertz wave energy and converts it into joule heat, which in turn exerts an effect on the structure and function of the neuron. Wilmink et al. (2011) investigated the thermal response of human skin dermal fibroblasts under terahertz irradiation and showed that the thermal effects of terahertz can be predicted by conventional bio-thermal methods. This provides a basis for modelling the thermal effects in neurons. Pennes (1948) built on this by considering the parameters of blood perfusion rate and metabolically-generated heat in biological tissues, and proposed the bio-thermal transfer equation:

$$\rho C_{p}\frac{\partial T}{\partial t}+\nabla ∙\left(-k\nabla T\right)=\rho_{b}C_{b}w_{b}\left(T_{b}-T\right)+Q_{met}+Q_{ext}$$ (6)

where ρ is the biological tissue density, $C_{p}$ is the thermal capacity of the biological tissue, T is the temperature distribution function, and k is the thermal conductivity of the biological tissue. The transfer term $Q_{pref}=\rho_{b}C_{b}w_{b}\left(T_{b}-T\right)$ represents convective cooling of the blood perfusion; $\rho_{b}$ is the blood density; $C_{b}$ is the specific heat capacity of the blood; $w_{b}$ is the blood perfusion rate; and $T_{b}$ is the temperature of the arterial blood. $Q_{met}$ is the power density generated by metabolism, which can be neglected in the calculation considering its small quantity in the neuron. $Q_{ext}$ is an external heat source. In the case of electromagnetic-thermal coupling, the heat generated by the electromagnetic wave in the neuron is $Q_{ext}$ . $Q_{ext}$ is transferred to the bio-thermal model during the simulation. In addition, little blood flows through the neurons, and the heat generated by metabolism is much less than that generated by electromagnetic waves. Thus, over the course of the calculation, the blood perfusion rate is neglected, and heat sources due to metabolism are ignored. The biological heat transfer equation can be simplified as:

$$\rho C_{p}\frac{\partial T}{\partial t}+\nabla ∙\left(-k\nabla T\right)=Q_{ext}$$ (7)

$$Q_{ext}=Q_{rh}+Q_{ml}$$ (8)

$$Q_{rh}=\frac{1}{2}Re\left(J∙E\right)$$ (9)

$$Q_{ml}=\frac{1}{2}Re\left(i\omega B∙H\right)$$ (10)

In solving the bioheat equation, the boundaries E and F are set to be thermally insulated, and the effect of heat dissipation from the cerebrospinal fluid is ignored. Considering that boundaries B, C, and D are distant from the light source region, we assume that the initial temperature is constant at the boundaries. The output parameters of the terahertz source, the initial conditions for solving the equations and the properties of the materials, are shown in Table 3.

### Fiber-coupled terahertz radiation platform and experimental protocol

The fiber-coupled terahertz radiation system used in the experiments was the TERA K15 from Menlo Systems, Germany, based on which the transmission optical path for terahertz has been improved. The effective frequency range of the terahertz system output is 0.1–2 THz, and the maximum output power is 100 μW. The system is transmitted through a fiber-optic connector to a fiber-coupled terahertz transmitter (TERA 15-TX-FC Fe: InGaAs) which generates terahertz waves under a bias voltage. The transmitter generates terahertz waves and can then focus the terahertz beam into a narrow space through four lenses (L1, L2, L3, L4). Since the terahertz waves generated by the transmitter diverges, the terahertz beam is first collimated by a plano-convex lens (L1); the longer the focal length of L1, the wider the beam’s diameter. The terahertz wave is then focused using a convex flat lens (L2) while placing the sample at its focal point. The plano-convex lens (L3) is used to collect any terahertz waves passing through the sample and collimate the terahertz beam, and the convex plane lens (L4) focuses the terahertz waves onto the detector to detect any terahertz waves passing through the sample.

To further investigate the pattern of terahertz wave interactions with neurons, we cultured neurons *in vitro*. Fetal mouse cortical neurons were first extracted and cultured for 2 days to allow cortical neurons to adapt to their environment and grow against the wall. To mitigate the absorption of terahertz waves by the culture medium, cortical neurons were radiated from the bottom of the Petri dish. Neurons cultured *in vitro* can be divided into 5 stages, with each stage measured in days. Therefore, the neurons were radiated using terahertz cumulative radiation, and to mitigate the thermal effect of terahertz radiation, the neurons were radiated for 3 min per day for 3 days.

### Experimental materials

SPF SD (Specific Pathogen Free Sprague Dawley) pregnant rats, at 12–15 days of gestation, were purchased from Beijing Vital River Laboratory Animal Technology Co., Ltd. The main experimental reagents used for cortical primary neuron culture were: Dulbecco’s Modified Eagle Medium (Gibco, 11965092), Neurobasal (Gibco, 21103049), B-27 (Gibco, 17504044), Fetal Bovine Serum (Gibco, 10099141C), Trypsin 0.25% (Gibco, 15050057), Penicillin-Streptomycin (Gibco, 15140163), Glutamine (Gibco, 35050061), Poly-L-lysine (Sigma, P4832), HBSS (Beynotime, C0218) and HEPES (Beynotime, ST090). After 4 h, the growing medium was replaced with a maintenance medium containing 97% Neurobasal, 2% B27% and 1% Glutamine. Two days later, the neurons were irradiated with terahertz for 3 min/day for 3 days.

### Primary neuron cultures

Primary neuronal culture was based on Guo et al. (2013) method, with slight modifications using SPF SD (Specific Pathogen Free Sprague Dawley) pregnant rats, 12–15 days of gestation, and bodyweight of 300–350 g. The fetal rats’ cerebral cortexes were extracted in a sterile bench, cut up, and added to Trypsin 0.25%, and then digested in an incubator for 15 min, and removed every 3 min. Slowly and gently, we blew the neuron with a flame-passivated pasteurized dropper. The cell suspension was grown in 10% fetal bovine serum diluted in 90% Dulbecco’s modified eagle medium and adjusted to a concentration of 1 × 10^4^ cells in 1 mL.

### Neuron structure parameter extraction and analysis method

Firstly, we ensured that each neuron recorded had been radiated by terahertz, and that we could find the same neuron quickly and efficiently. Two marker lines were drawn on the Petri dish, and the intersection of the two lines was at the center of the Petri dish. The neuronal growth and development was photographed in the four quadrants with the center of the Petri dish as a right-angle coordinate system. Next, images with a clear background and low neuronal density (where the cell bodies and protrusions of individual neurons can be observed and are not connected to other neurons) were selected. Then, we used ImageView to open the optical microscope to take pictures, and used any connection curve in the “Measure” menu to measure the neurite protrusion length, and any polygon to measure the neurite cell area. The total neuronal protrusion length growth value and the cell body area growth value were calculated as follows:

$${∆Neurites}_{i}^{j}=\frac{\sum_{k=1}^{n}{Neurites}_{i}^{j}\left(k\right)-{Neurites}_{i}^{0}\left(k\right)}{n}$$ (11)

$${∆Soma}_{i}^{j}=\frac{\sum_{k=1}^{n}{Soma}_{i}^{j}\left(k\right)-Soma}{n}$$ (12)

where ${∆Neurites}_{i}^{j}$ denotes the average growth value of the total length of neuronal protrusions in the *i*th culture dish after the *j*th day of terahertz radiation. ${Neurites}_{i}^{j}\left(k\right)$ denotes the total length of the *k* neuronal protrusion in the *i*th petri dish after the *j*th day of terahertz radiation. ${Neurites}_{i}^{0}\left(k\right)$ denotes the total length of the *k* neuronal protrusion in the *i*th petri dish before terahertz radiation. Similarly, Soma denotes the area of the neuron’s cytosol. The neuronal developmental state was recorded before terahertz radiation, and the neuronal cytosolic area and total protrusion length at this point were used as initial values. The total length of the neuronal cell area and the protrusion after 24 h of the first terahertz radiation was recorded and subtracted from the initial value as the growth of neuronal cell area and protrusion on the first day after terahertz radiation. The growth values of the neuronal cytosolic area and total protrusion length after the second and third day after terahertz radiation were calculated in the same way, respectively. Finally, the neuronal parameters measured in the same culture dish were averaged to represent the cellular condition of the entire dish, and used as a sample value.

### Quantification and statistical analysis

All data are expressed as mean ± standard error of the mean (SEM), with specific cases noted separately. Firstly, the samples are tested for normality. Secondly, significance analysis of the results of the experimental and control groups was performed using an independent samples *t*-test. Statistical analysis was done by MATLAB, and results were considered significantly different when *p* < 0.05.

## Results

### The propagation law of terahertz waves in neurons

**FIGURE 2:**
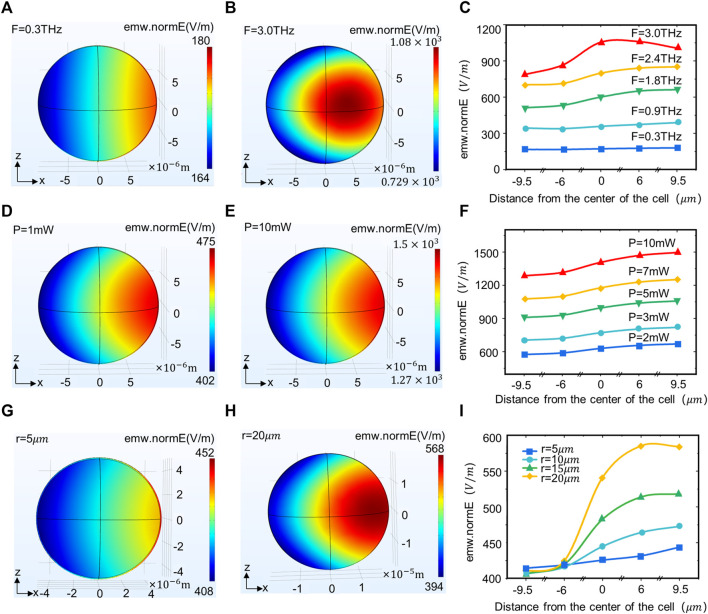
Terahertz wave propagation in neurons with different terahertz radiation parameters and neuron size. **(A, B)** Propagation law of terahertz waves with frequencies of 0.3 and 3 THz in the XZ plane of neurons. **(C)** Propagation law of terahertz waves with different powers in neurons (simulation time: steady state, initial temperature: 293.0 k, r = 10 um, F = 0.3, 0.9, 1.8, 2.4, 3.0 THz, *p* = 1 mW). **(D**, **E)** Propagation law of terahertz waves with Power of 1 and 10 mW in the XZ plane of neurons. **(F)** Propagation law of terahertz waves with different powers in neurons (simulation time: Steady state, initial temperature: 293.0 k, r = 10 um, F = 1.2 THz, *p* = 2, 3, 5, 7, 10 mW). **(G, H)** The propagation law of terahertz waves in different neuron sizes (radii 5 and 10 um) in the XZ plane. **(I)** Propagation law of terahertz waves in neurons of different sizes (simulation time: Steady state, initial temperature: 293.0 k, r = 5, 10, 15, 20 um, F = 1.2 THz, *p* = 1 mW).

The neuron size and terahertz wave wavelength are both in the micrometer range and have the conditions for an interaction. Therefore, we investigated the correlation between different terahertz radiation parameters and cell size with the field strength distribution in neurons. The results show that when terahertz is radiated to the neuron, the energy is lower on the left side (the side where the terahertz enters the neuron) and gradually increases along the axis (Figures 2A, B). As the frequency of the terahertz waves increases, the energy at the same location in the neuron continues to rise. Meanwhile, the location of the energy maximum in the neuron gravitates towards the center of the neuron (Figure 2C). When the terahertz radiation power changed, the energy distribution in the neuron did not change significantly (Figures 2D, E), but the energy in the same location in the neuron kept rising (Figure 2F). There is also variation in neuron size across functions and regions of an organism, and we found that as neuron size increases, the energy in the center and to the right of the neuron continues to rise (Figures 2G–I).

### Thermal effects patterns in terahertz wave interactions with neurons

**FIGURE 3:**
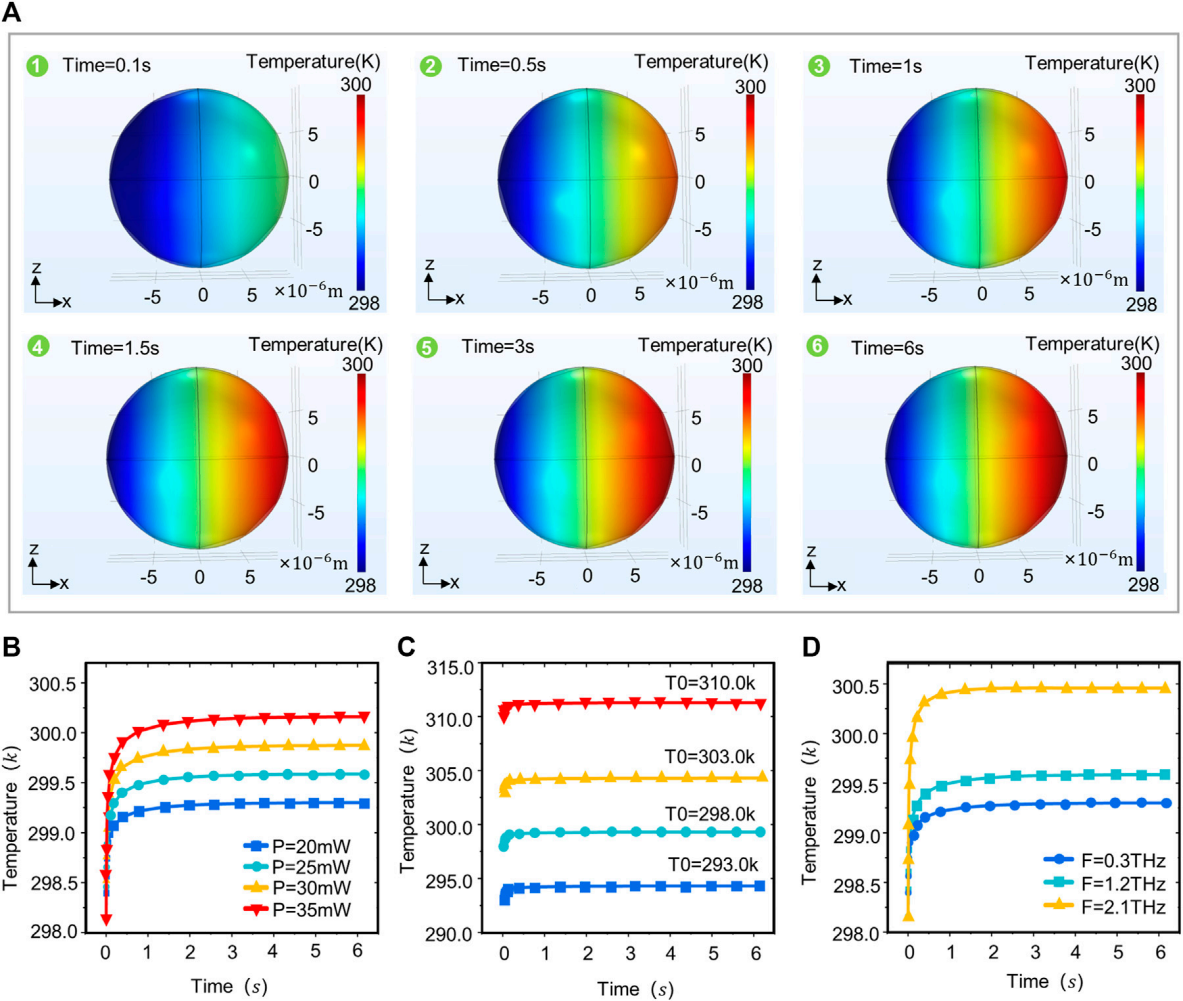
Temperature distribution patterns in neurons under different terahertz radiation parameters. **(A)** The XZ plane temperature of neurons varies with terahertz radiation time (Simulation time: 6 s, Initial temperature: 293.0 k, r = 10 um, F = 1.2 THz, *p* = 20 mW). **(B)** Variation of temperature in neurons with time at different terahertz radiation powers (simulation time: 6 s, initial temperature: 293.0 k, r = 10 um, F = 1.2 THz, *p* = 20, 25, 30, 35 mW). **(C)** Variation of temperature in neurons with time at different initial temperatures (simulation time: 6 s, initial temperature: 293.0, 298.0, 303.0, 310.0 k, r = 10 um, F = 1.2 THz, *p* = 20 mW). **(D)** Temperature variation in neurons with time at different terahertz radiation frequencies (simulation time: 6 s, initial temperature: 293.0 k, r = 10 um, F = 0.3, 1.2, 2.1 THz, *p* = 20 mW).

The thermal effect of terahertz waves is due primarily to the absorption of terahertz waves by neurons and their conversion into thermal energy (Liu GZ. et al., 2019). The absorption properties of terahertz waves by neurons are associated primarily with water molecules and biomolecules. But high temperatures can exert some negative effects on neurons, and thus we investigated the correlation between terahertz radiation parameters and changes in neuronal temperature (Li et al., 2021). The results show that the temperature distribution pattern in the neuron is similar to the field strength distribution pattern (Figure 3A), with lower temperatures on the left side of the neuron (the side where the terahertz wave enters the neuron). The temperature in the neuron increases continuously along the axis, peaking on the right side of the neuron (the side through which the terahertz wave passes). The temperature in the neuron rapidly stabilizes within 1 s, and spikes within 0–0.5 s. The time to reach temperature stability in the neuron is independent of the initial temperature, terahertz radiation power and frequency (Figure 3B). As the power of the terahertz radiation increases, the temperature in the neuron continues to increase (Figure 3B). The temperature in the neuron varies with the time of terahertz radiation in the same pattern for different initial temperatures (Figure 3C). The frequency of terahertz waves is also a major influence on the temperature in the neurons, which is positively correlated with the terahertz wave frequency (Figure 3D).

### Terahertz radiation promotes neuronal cytosol and protrusion growth

**FIGURE 4:**
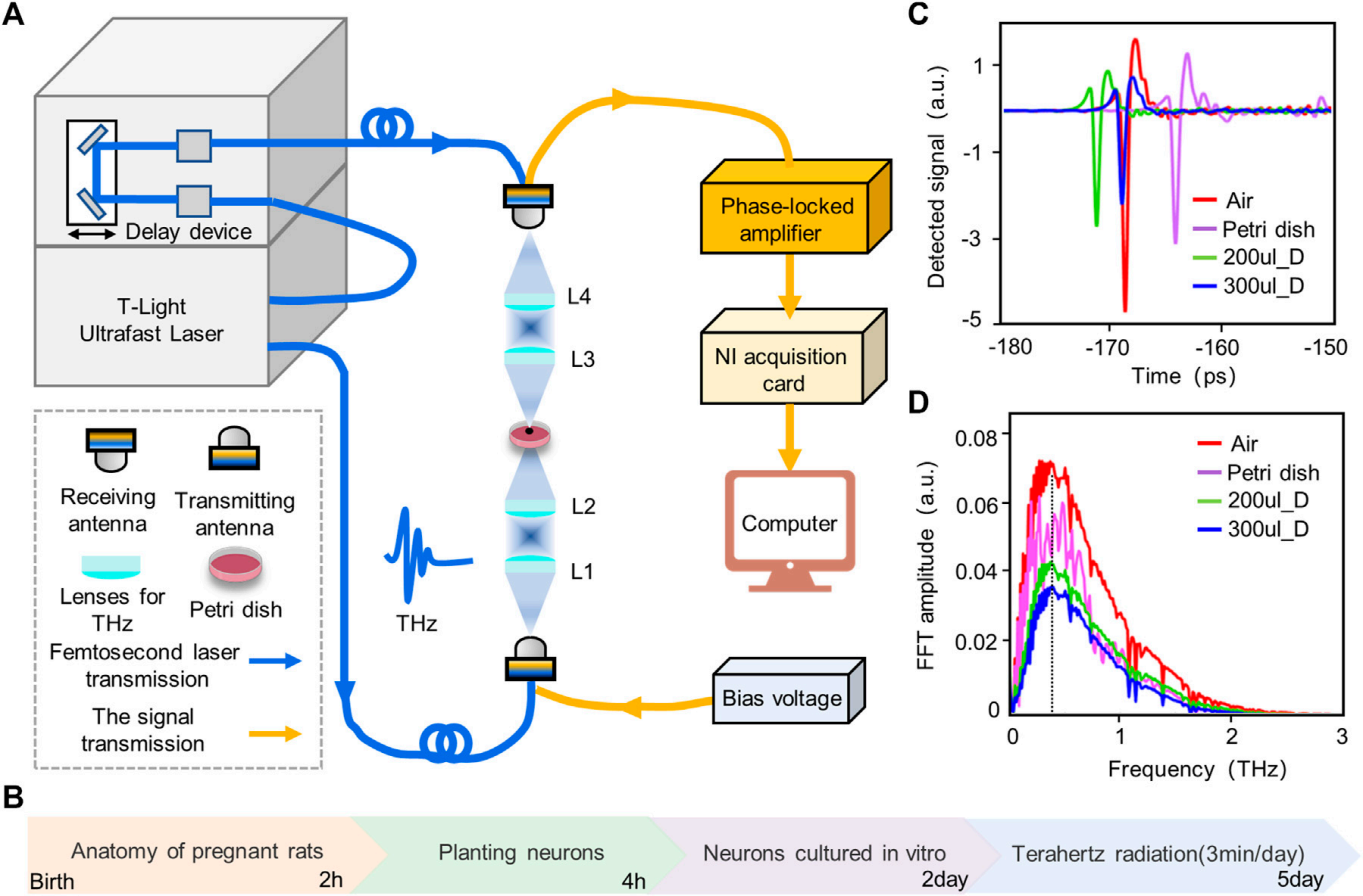
Experimental platform, protocol and terahertz wave attenuation test. **(A)** Experimental platform for wide-band terahertz radiation of cortical neurons. We radiated neurons from the bottom of a petri dish and measured the amplitude and frequency of the terahertz waves passing through the dish in real time. **(B)** Experimental protocol for terahertz radiating neurons. The experiment lasted for 5 days, with neurons being planted and cultured for the first 2 days, followed by the accumulation of radiated neurons for 3 consecutive days. **(C)** Time domain diagram of terahertz after penetrating air, petri dish, 200 ul Dulbecco’s modified eagle medium (200 ul_D) and 300 ul Dulbecco’s modified eagle medium (300 ul_D). **(D)** Frequency domain map of terahertz after penetrating air, petri dish, 200 ul_D and 300 ul_D.

At the beginning of the study, due to the large absorption of terahertz waves by the cell culture medium, the time and frequency domain signals of the terahertz waves after penetrating the empty culture dish and culture medium were measured in order to estimate the energy of the terahertz radiation to neurons and the frequency band range. Terahertz radiation system and radiation protocol are shown in Figures 4A, B. The results show that the terahertz waves can penetrate the culture dish and the culture fluid; the frequency range of the transmitted waves is 0.1–2 THz; and the main energy is concentrated in 0.3–1 THz (Figures 4C, D). At the same time, the absorption of terahertz by the culture fluid is large, and the energy of the transmitted wave is negatively correlated with the volume of the culture fluid.

**FIGURE 5:**
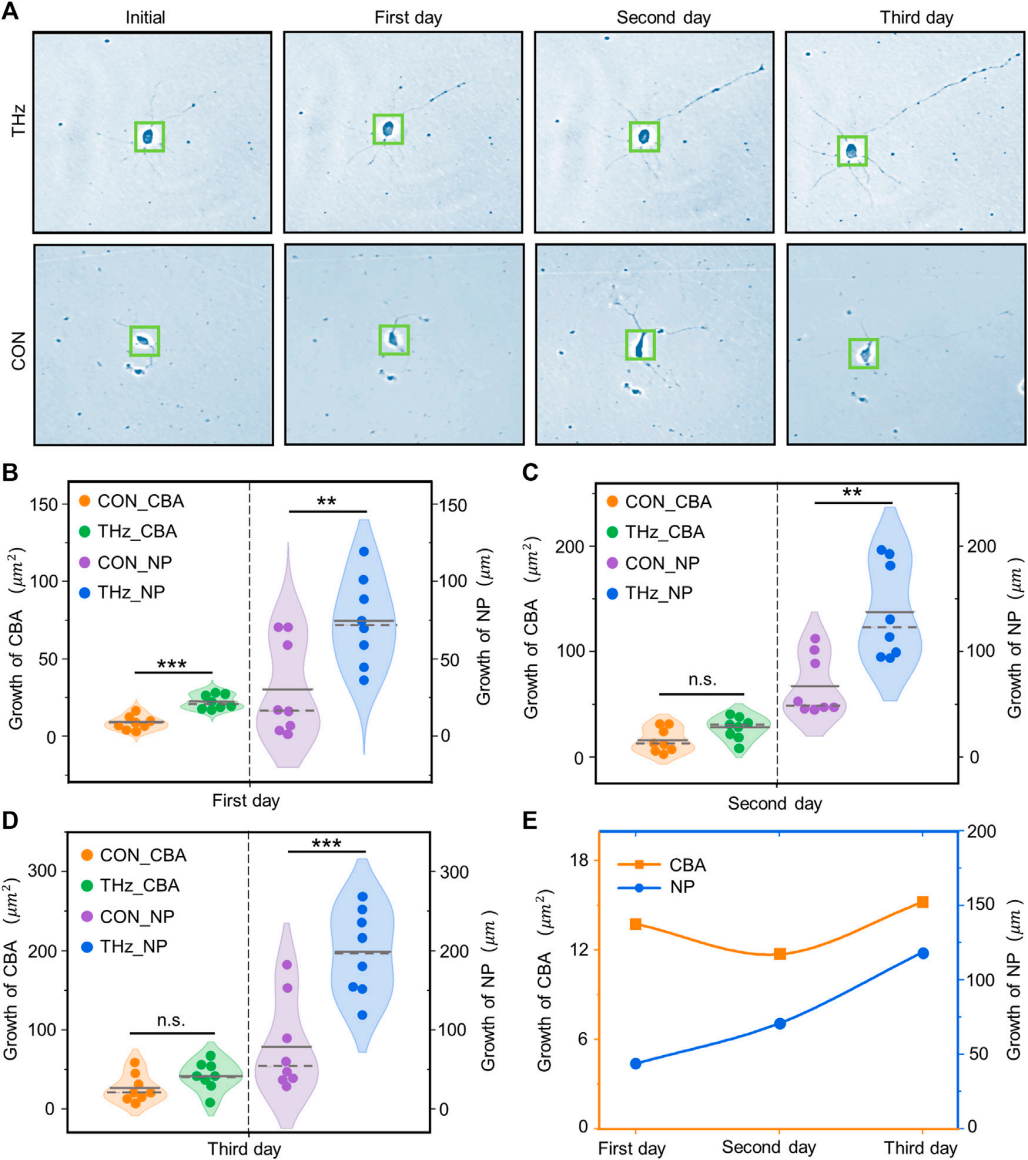
Broadband terahertz radiation promotes the dynamic growth and development of cortical neurons. **(A)** Representative performance maps of neuronal developmental status in the Terahertz (THz) and Control (CON) groups. **(B–D)** Significance analysis of the difference between THz and CON in terahertz cumulative radiation neurons for 3 days. On the first day, neurite protrusion (NP) and cell body area (CBA) were significantly higher than the CON. On the second day NP was significantly higher than the CON, but CBA showed no significant difference. On the third day, there was no significant difference between CBA and CON, but NP was significantly higher than the CON. Data are mean ± SEM (n = 8 independent experiments). **, *p* < 0.01). ***, *p* < 0.001 compared with CON, independent samples *t*-test. **(E)** Relationship between the radiation days in THZ_CBA minus CON_CBA and THZ_NP minus CON_NP.

To investigate the effect of terahertz radiation on neuronal growth and development, the neuronal cytosolic area and total protrusion length were used as statistical analysis quantities. Neuronal protrusions in the control and terahertz groups began to grow after 1 day of terahertz radiation, and subsequently showed neuronal properties that remained well-developed after the end of terahertz radiation (Figure 5A). The neuronal cytosol is the center of neuronal metabolism and nutrition, and is associated with neuronal survival and development (Abrahamsson et al., 2017; Yang et al., 2017). We found that the growth value of the neuronal cytosolic area in the control group increased as number of days increased, and the same phenomenon was found in the terahertz group. We further statistically analyzed the effect of terahertz radiation on neuronal cytosolic growth. The growth value of the neuronal cell area was significantly higher in the terahertz group (22.6 ± 1.6 ${\mu m}^{2}$ ) than in the control group (8.9 ± 1.5 ${\mu m}^{2}$ ) on Day 1 (Figure 5B). The terahertz group was higher than the control group on Days 2 and 3, but the statistical results were not significant (Figures 5C, D).

The protrusions of neurons are the basis for information communication between neurons, and are the key to the formation of neural networks (Moore et al., 2020). In the control group, the growth value of the total neuronal protrusion length showed a positive correlation with the number of days, and the same phenomenon was present in the terahertz group. The value of total neuronal protrusion length growth was significantly higher in the terahertz group (74.2 ± 10.0 μm ) than in the control group (30.6 ± 10.9 μm ) on Day 1 of terahertz radiation (Figure 5B). Additionally, the terahertz group (138.1 ± 16.0 μm , 197.8 ± 19.0 μm ) was significantly higher than the control group (67.7 ± 10.1 μm , 79.5 ± 20.1 μm ) on Days 2 and 3 of terahertz radiation (Figures 5C, D). In addition, we have analyzed the relationship between the radiation days in THZ_CBA (Terahertz radiation_Cell Body Area) minus CON_CBA (Control_Cell Body Area) and THZ_NP (Terahertz radiation_Neurite Protrusion) minus CON_NP (Control_Neurite Protrusion). We found that THZ_NP minus CON_NP is positively correlated with the number of days of radiation, but THZ_CBA minus CON_CBA is non-linear with the number of days of radiation (Figure 5E).

## Discussion

Neuron sizes and terahertz wave wavelengths are on the order of microns and can interact with each other. Due to the disparity in relative permittivity between the cell membrane and the cytoplasm, a large amount of reflected signal is generated when terahertz waves enter the cell membrane from the cytoplasm. The reflected signals are superimposed on each other near the boundary of the two substances, resulting in a standing wave effect (Liu WQ. et al., 2019). Thus, the field strength and temperature in the neuron show up as low on the left side (the side where the terahertz wave enters the neuron) and high on the right side (the side where the terahertz wave transmits out of the neuron). This phenomenon is influenced by the wavelength of the terahertz wave and the size of the neuron, and does not occur when the neuron size is either much smaller or much larger than the terahertz wavelength. In addition, the field strength and temperature in the neuron are positively correlated with the frequency and power of the terahertz wave. This phenomenon is related to the relative dielectric constant of the cytoplasm. The main component of the cytoplasm is water, which can be regarded as a polar fluid, and most of its losses are polarized losses (Generalov et al., 2020). As the terahertz frequency increases, the cytoplasm’s polarization loss attenuates and the neuron’s internal field strength and temperature increase.

Individual terahertz photons have energies as low as meV (milli-electron volts), and do not have a direct ionizing damaging effect similar to that of x-rays (Xiang et al., 2019). However, as the terahertz beam intensity increases, terahertz waves cause a range of biological effects on neurons. The thermal effect of terahertz waves is mainly due to the absorption of terahertz waves by neurons and their conversion into thermal energy. The absorption properties of terahertz waves by neurons are associated primarily with water molecules and biomolecules. Since neurons contain much more water than biomolecules, and are the main chromophore in the terahertz band in neurons, the thermal effect produced is due primarily to water. Water has many unique properties in that its molecules can form hydrogen bonds with neighboring water molecules, creating a dynamic hydrogen bonding network (Yada et al., 2008). The intermolecular stretching and bending of vibrational modes of this network are in the terahertz band (strong resonance frequency of 1.5 THz). When a neuron is exposed to terahertz wave radiation, the vibrational modes of the hydrogen bonds in it are excited, thus triggering resonance (Duan et al., 2021). The dynamic equilibrium of the water molecular network structure is broken, resulting in strong absorption of terahertz waves by the water (Kristensen et al., 2016). The absorbed terahertz wave energy is converted into kinetic energy for the water molecules’ own irregular motion, and the frequency of mutual collisions increases. This generates heat energy (Heshmat, et al., 2017; Balos et al., 2022). In the absence of photochemical processes and phase changes, this will lead directly to an increase in neuronal temperature as heat collects.

The thermal effect of terahertz radiation on neurons leads to changes in neuron structure and function (Zhang et al., 2021). This change has two primary causes: The magnitude of the neuronal temperature increase and the duration of the high temperature. When neurons are exposed to high temperatures for long periods, this can lead to disturbed neuronal growth, dehydration effects, neuronal morphological damage, neuronal stress responses, and more seriously, structural protein degeneration and neuronal death (Samsonov and Popov, 2013). Moreover, when high temperatures in neurons persist at the millisecond level, they have reversible effects on neurons (affecting intra-neuronal calcium homeostasis, inducing action potentials in neurons, and affecting neuronal synaptic transmission and neuronal firing rates) (Albert et al., 2012; Fribance, et al., 2016).

When studying terahertz neurobiological effects, it is first necessary to consider the safety of the terahertz radiation protocol. Safe terahertz radiation parameters ensure both that sufficient power is radiated to the neuron and that the temperature effect on the neuron is mitigated. Thus, field strength and temperature in neurons can characterize (to an extent) the terahertz radiation parameters’ safety. From a physical point of view, the biological effects of terahertz wave radiation on neurons are derived from the thermal and non-thermal effects of terahertz waves (Peng and Zhou, 2021). Therefore, the temperature variation in neurons needs to be reduced when selecting terahertz radiation parameters to facilitate distinguishing whether the neuronal response is caused by thermal or non-thermal effects. Usually, we are looking at the non-thermal effects of terahertz waves, so the parameters are chosen by first determining the range of permissible temperature increases in the neurons. This temperature range ensures that there is no effect on the structure and function of the neurons. The non-thermal effect arises mainly from the non-linear resonance effect of biological macromolecules in neurons excited by terahertz waves (Peng and Zhou, 2021). The choice of frequency for terahertz waves is crucial because of the different resonance peaks between biological macromolecules. The terahertz wave frequency can be determined based on the modulating substance’s properties. The terahertz radiation’s power is then determined, and an appropriate reduction in power will mitigate the rise in temperature in the neurons. When we need to use more radiation power, we can use pulsed waves, and controlling the duration of a single round of radiation to milliseconds can also mitigate the rise in temperature in the neurons. When the temperature in the neurons cannot be mitigated by regulating the terahertz radiation protocol, the biological sample can be placed in an automatic temperature control system (Zhao et al., 2021). The effects of temperature changes on neurons can also be mitigated if the temperature control system’s accuracy and adjustment time are sufficient. Alternatively, we could use short periods of cumulative radiation.

On this basis, we have investigated the effects of Terahertz wave cumulative radiation on neuronal structures. To mitigate the thermal effects of terahertz radiation on neurons, broadband trace amounts of terahertz served as the radiation source (frequency band 0.1–2 THz, maximum radiation power 100 μW), and short periods of cumulative radiation were used (3 min/day, 3 days). We found that this radiation protocol causes no significant negative effects on the neurons. Additionally, we found significantly higher neuronal cell area growth values after the first day of terahertz radiation, compared to the control group. However, there were no significant differences on either the second or the third days. We also found that the total length of the neuronal protrusions had increased by 94.6% on Day 1, 76.0% on Day 2, and 100.0% on Day 3, compared to the control group. These results were all statistically significant. The reasons for these phenomena may be related to the developmental cycle of cultured neurons *in vitro*. It has been shown that there is a “latency period” after inoculation of neurons in *in vitro* cultures, during which cells adhere to the wall and adapt to their environment, with slow cell growth. At the end of the incubation period, the cells enter a rapid growth phase in which the cytosol and protrusions grow rapidly, a phenomenon that generally occurs between Days 2 and 5 of cell inoculation. The protrusions rapidly (within 5–7 days of cell inoculation) develop into dendrites, while the axons maintain rapid growth as well (Sulatsky, et al., 2014; Cha, 2016). The first day of terahertz radiation coincided with the third day of neuronal inoculation, when the cells were in a period of rapid growth, and the neuronal cytosolic growth rate was significantly higher after terahertz radiation than in the control group. At the same time, neuronal protrusions remained in a rapid growth phase during the 3 days of terahertz radiation. Thus, the neuronal protrusions’ growth rate was significantly higher after these 3 days of terahertz radiation than in the control group. These findings suggest that terahertz radiation-promoted neuronal growth only occurs during periods of intrinsic neuronal growth.

## Conclusion

In this study, the propagation and thermal effects of terahertz waves’ interactions with neurons were modelled to analyze the main parameters affecting the field strength and temperature variation in terahertz waves in neurons. The field strength and temperature variations are used as evaluation criteria to select the appropriate terahertz radiation parameters. On this basis, we experimentally investigated the effects of cumulative radiation from terahertz waves on the structure of neurons. 1) The frequency and power of terahertz waves are the main factors affecting field strength and temperature in neurons, and there is a positive correlation between them, a phenomenon that is correlated with the relative dielectric constant of the cytoplasm. 2) When choosing terahertz radiation parameters, the frequency can be determined according to the properties of the modulating substance, and an appropriate reduction in radiation power can mitigate the rise in temperature in the neuron. It is also possible to use pulsed waves to keep the duration of a single round of radiation, as well as short bursts of cumulative radiation, to the millisecond level. 3) On this basis, we found that broadband trace terahertz (0.1–2 THz, maximum radiated power 100 μW), short duration cumulative radiation (3 min/day, 3 days) does not cause neuronal death. This radiation protocol can also promote the growth of neuronal cytosomes and protrusions. This paper provides a set of guidelines and methodology for selecting terahertz radiation parameters in the study of terahertz neurobiological effects. Furthermore, it provides verification that the structure of neurons can be modulated using short duration cumulative radiation.

## Data Availability

The raw data supporting the conclusion of this article will be made available by the authors, without undue reservation.
